# Pneumomediastinum After Dental Filling: A Rare Case Presentation

**DOI:** 10.7759/cureus.5593

**Published:** 2019-09-08

**Authors:** Robert D Rawlinson, Ulugbek Negmadjanov, David Rubay, Levonti Ohanisian, Jonathan Waxman

**Affiliations:** 1 Surgery, Charles E. Schmidt College of Medicine, Florida Atlantic University, Boca Raton, USA; 2 Orthopaedic Surgery, Charles E. Schmidt College of Medicine, Florida Atlantic University, Boca Raton, USA; 3 Thoracic Surgery, Charles E. Schmidt College of Medicine, Florida Atlantic University, Boca Raton, USA

**Keywords:** pneumomediastinum, subcutaneous emphysema

## Abstract

Pneumomediastinum and subcutaneous emphysema is an uncommon potentially life-threatening complication of dental procedures. Common causes of pneumomediastinum after dental procedures include tooth extraction, preparation, restorative treatment, endodontic treatment, and subgingival curettage that are associated with the use of handpieces and high-pressure air/water syringes. Herein, we present a case of pneumomediastinum with subcutaneous emphysema in a 40-year-old female who underwent two dental fillings and presented to our hospital with chief complain of facial swelling and odynophagia. The patient was managed conservatively, had an uneventful hospital course, and fully recovered. This case underlines the need for prompt diagnosis and management because of the risk of airway compromise, air embolism, and infection. The mechanism, clinical presentation, differential diagnosis, and complications are also reviewed.

## Introduction

Cervicofacial emphysema and pneumomediastinum are uncommonly observed complications of dental interventions. The complications are usually associated with the use of a high-speed air-turbine dental drill on second and third lower molars as the roots of these teeth communicate directly with the sublingual and submandibular space. The air from the drill is thought to pass through those spaces to the pterygomandibular, parapharyngeal, and retropharyngeal spaces as well as mediastinum. Typical presenting scenarios are associated with more invasive oral and endodontic procedures, including teeth extraction and root canals. Due to the risk of bacterial spread between the maxillofacial fascial space and mediastinum, this could potentially lead to life-threatening infections of the retropharyngeal space and mediastinum [1]. However, the majority of cases are usually self-limiting and benign. Thoracic surgeons are involved in the diagnosis and management of this entity because of the potentially life-threatening conditions such as esophageal perforation or necrotizing mediastinitis that either must be treated as an emergency or excluded.

## Case presentation

**Figure 1:**
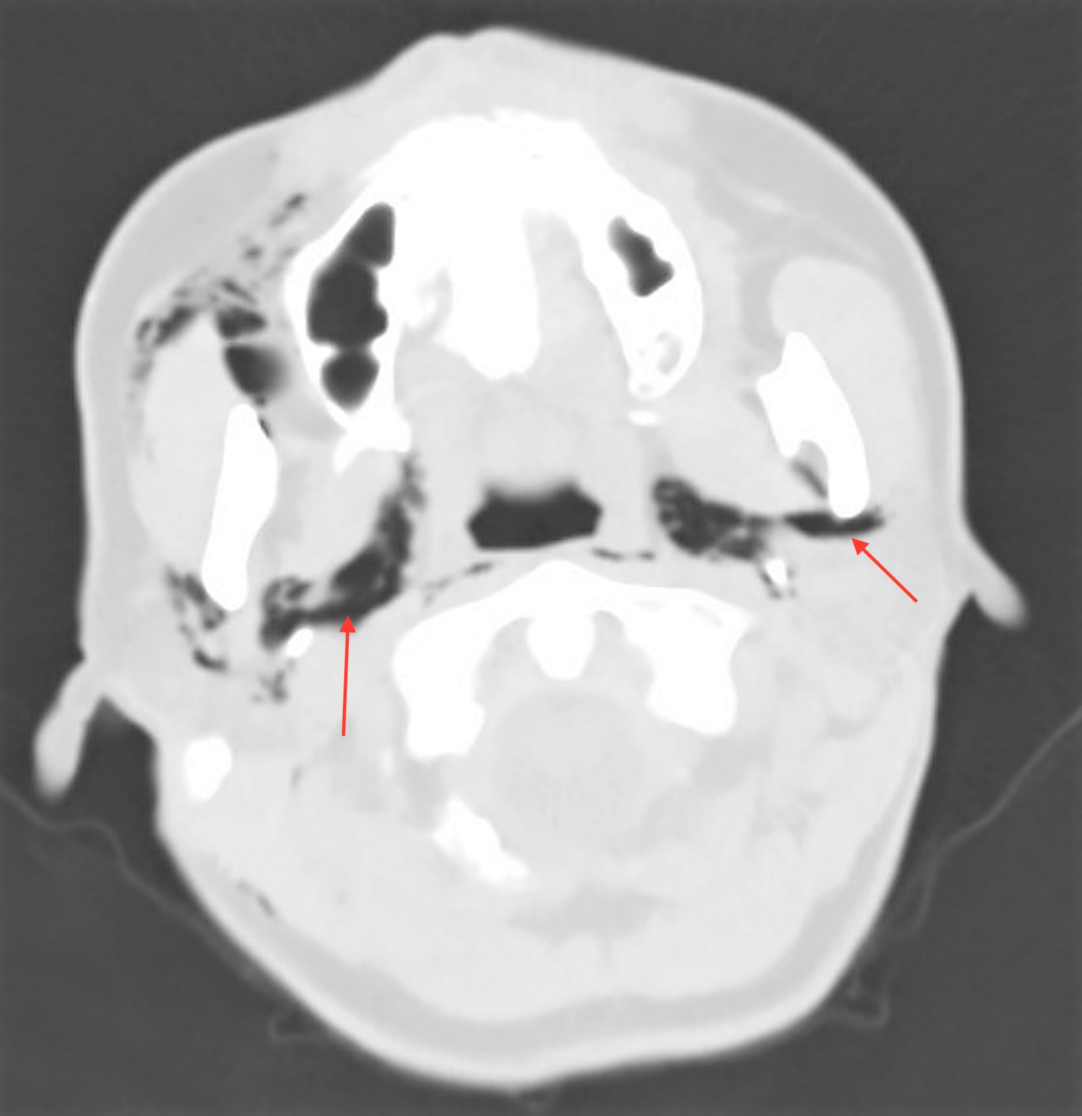
Axial CT image of facial soft tissues with air in the carotid and retropharyngeal space.

**Figure 2:**
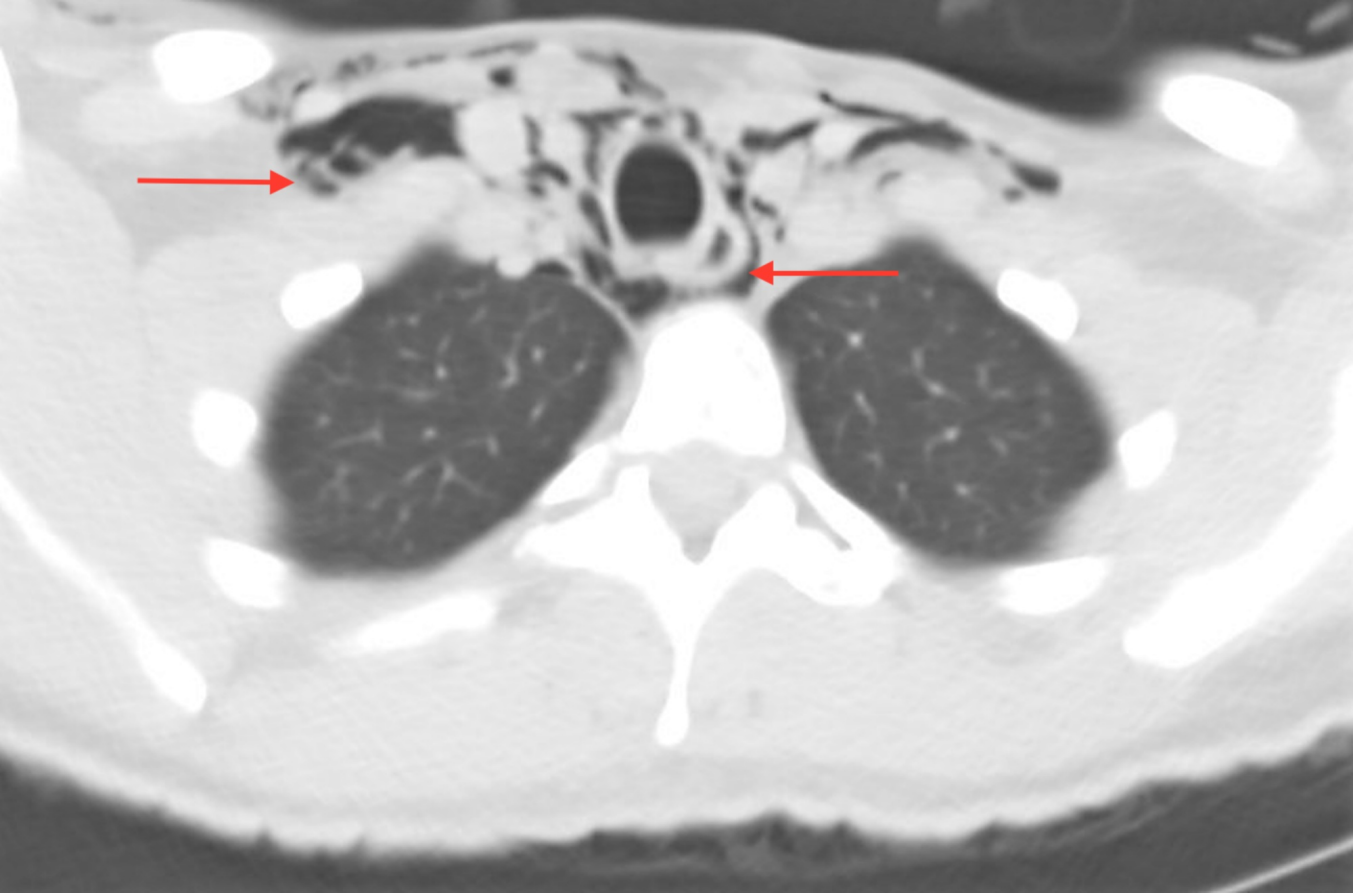
Axial CT image of the superior mediastinum.

**Figure 3:**
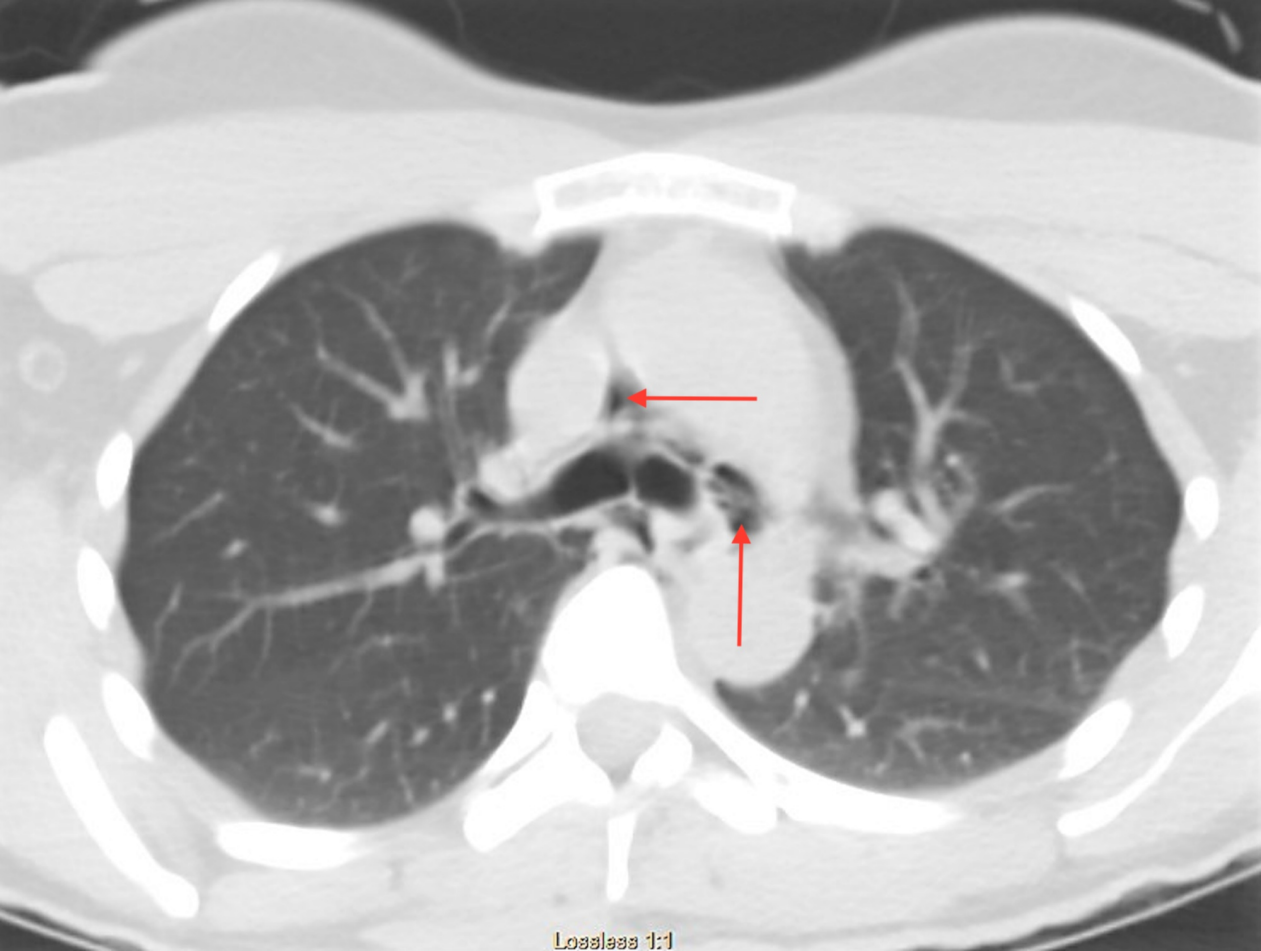
Axial CT of the thorax with air seen around the major bronchi.

A 40-year-old female presented to the ED complaining of facial swelling, bleeding, and odynophagia after undergoing dental fillings earlier the same day. Earlier that day, the patient reported that she had dental fillings completed on the upper left and lower right molar teeth. On evaluation, she was afebrile and hemodynamically normal, and on physical examination, she was hemodynamically stable without evidence of respiratory compromise. The patient did have right-sided facial swelling and crepitus extending down the neck without skin discoloration. Her lung fields were clear to auscultation, and she did not have any adenopathy. Initial laboratory evaluation was significant for a leukocytosis of 12,500/mcL. Computed tomography (CT) of the facial soft tissues and chest demonstrated extensive subcutaneous emphysema and pneumomediastinum extending from the lower neck and surrounding the trachea and upper lobe bronchi (Figures 1-3). The patient was transferred to the ICU for close observation, where she was started on broad-spectrum intravenous antibiotics and intravenous fluids. Thin barium esophagogram was performed, without evidence for perforation. After 24 hours, the patient’s facial swelling improved, and she was discharged home. Her follow-up was unremarkable.

## Discussion

Subcutaneous emphysema and to a lesser extent pneumomediastinum have been reported after dental procedures going back 100 years ago. Arai et al. reviewed 47 cases of CT-documented cases of subcutaneous emphysema with or without pneumomediastinum between 1994 and 2008 [2]. In the reported cases, the most common procedure leading to subcutaneous emphysema was tooth extraction and dental fillings, particularly in the mandibular third molar. Routine use of high-speed rotation drills may introduce air from the oral cavity by disrupting the mucosa and periodontal ligament. Mucosal disruption is most commonly the result of trauma, or as in our case, iatrogenic injury. Air introduced, especially under pressure, can dissect through the fascial planes of the neck into the mediastinum [3-4]. The deep cervical fascia divides the neck into three compartments: the perivisceral space anteriorly which is separated from the visceral space by the pretracheal fascia, and the prevertebral space posteriorly. The visceral space invests the trachea and esophagus continuing directly into the chest and can create a direct conduit for the free movement of gas between the neck and mediastinum. The visceral space inferiorly is continuous with adventitia of the great vessels and major airways and follows the esophagus through the diaphragmatic hiatus into the retroperitoneal soft tissue space. Infection is not usually observed in these cases, and surgical treatment is not usually needed. However, the potential risk of iatrogenic emphysema caused by using air-powder abrasive devices should be recognized. There is a potential risk for infection, considering the frequent use of air-powder abrasive devices to treat peri-implantitis. Other infectious etiologies typically result from spreading from oral, pharyngeal, or tonsillar abscesses or even osteomyelitis of facial bones or salivary gland infection. Esophageal rupture due to profuse emesis can also cause suppurative infection of the mediastinum and is a devastating complication. Spontaneous etiology is rarer but has been described following extreme effort or Valsalva as during childbirth [2], it is more commonly associated; however, with ventilator-associated barotrauma.

Early recognition and diagnosis with appropriate management are paramount. While spontaneous improvement is expected and conservative management mostly indicated, there are serious complications that can arise and that treating providers must be cognizant of. Air accumulation in the parapharyngeal and retropharyngeal spaces can lead to airway compromise. Deep tracking of subcutaneous emphysema can lead to air embolism or pneumothorax. Infectious etiologies should be ruled out but, the emphysema, primarily if related to an intraoral etiology, can lead to secondary infection [5]. For these reasons, it is recommended to observe the patient and ensure the emphysema with or without pneumomediastinum does not worsen, the respiratory status remains uncompromised, administer antibiotics, and closely follow-up the patient.

## Conclusions

Subcutaneous emphysema and pneumomediastinum encountered in the ED by surgical trainees can be easily diagnosed based on palpable crepitus, physical exam, and detailed history. When encountered, the typical suspects are esophageal perforation, lung injury, bullae rupture, traumatic injuries to the chest as well as endoscopic and surgical procedures. In these cases, the etiology should be identified, and prompt management is necessary to prevent adverse events. Though subcutaneous emphysema and pneumomediastinum secondary to dental instrumentation is a rare entity that has lower morbidity, increased awareness should be present amongst surgical trainees when evaluating these patients to avoid unnecessary workup and invasive interventions.
